# Improving Vesicular Integrity and Antioxidant Activity of Novel Mixed Soy Lecithin-Based Liposomes Containing Squalene and Their Stability against UV Light

**DOI:** 10.3390/molecules25245873

**Published:** 2020-12-11

**Authors:** Sahar Pakbaten Toopkanloo, Tai Boon Tan, Faridah Abas, Mohammad Azam, Imededdine Arbi Nehdi, Chin Ping Tan

**Affiliations:** 1 Department of Food Technology, Faculty of Food Science and Technology, Universiti Putra Malaysia, Serdang 43400, Selangor, Malaysia; pakbaten.toopkanloo@gmail.com; 2 Department of Food Service and Management, Faculty of Food Science and Technology, Universiti Putra Malaysia, UPM, Serdang 43400, Selangor, Malaysia; taiboon_tan@upm.edu.my; 3 Department of Food Science, Faculty of Food Science and Technology, Universiti Putra Malaysia, UPM, Serdang 43400, Selangor, Malaysia; faridah_abas@upm.edu.my; 4 Chemistry Department, College of Science, King Saud University, P.O. Box 2455, Riyadh 11451, Saudi Arabia; mhashim@ksu.edu.sa (M.A.); imed12002@gmail.com (I.A.N.); 5 Chemistry Department, El Manar Preparatory Institute for Engineering Studies, Tunis El Manar University, P.O. Box 244, Tunis 2092, Tunisia; 6 Laboratory of Processing and Product Development, Institute of Plantation Studies, Universiti Putra Malaysia, Serdang 43400, Selangor, Malaysia

**Keywords:** liposome integrity, squalene, membrane composition, photodegradation, X-ray diffraction

## Abstract

In order to improve the membrane lipophilicity and the affinity towards the environment of lipid bilayers, squalene (SQ) could be conjugated to phospholipids in the formation of liposomes. The effect of membrane composition and concentrations on the degradation of liposomes prepared via the extrusion method was investigated. Liposomes were prepared using a mixture of SQ, cholesterol (CH) and Tween80 (TW80). Based on the optimal conditions, liposome batches were prepared in the absence and presence of SQ. Their physicochemical and stability behavior were evaluated as a function of liposome constituent. From the optimization study, the liposomal formulation containing 5% (*w*/*w*) mixed soy lecithin (ML), 0.5% (*w*/*w*) SQ, 0.3% (*w*/*w*) CH and 0.75% (*w*/*w*) TW80 had optimal physicochemical properties and displayed a unilamellar structure. Liposome prepared using the optimal formulation had a low particle size (158.31 ± 2.96 nm) and acceptable %increase in the particle size (15.09% ± 3.76%) and %trolox equivalent antioxidant capacity (%TEAC) loss (35.69% ± 0.72%) against UV light treatment (280–320 nm) for 6 h. The interesting outcome of this research was the association of naturally occurring substance SQ for size reduction without the extra input of energy or mechanical procedures, and improvement of vesicle stability and antioxidant activity of ML-based liposome. This study also demonstrated that the presence of SQ in the membrane might increase the acyl chain dynamics and decrease the viscosity of the dispersion, thereby limiting long-term stability of the liposome.

## 1. Introduction

When carrier complexes are applied as oral delivery systems in the food and nutraceutical industries, it is imperative that they must be stable in food formulations and are non-toxic and biodegradable. Liposomes resemble the structure of biomembranes and are suitable for use as a delivery system in food to ensure good cellular uptake [1]. However, they are also known to be unstable against external and internal stresses including physical (e.g., temperature, light), chemical (e.g., redox, pH) and biological (e.g., enzymes) [2,3]. Thus, for an efficient application of liposomal carriers as food and nutraceutical delivery systems, it is necessary to obtain deeper insights into the impact of incorporated materials on membrane integrity and vesicle stability by modulating the composition of the lipid bilayer.

Mixed soy lecithin (ML) has been widely used in fabricating liposomes. However, the liposomes from ML were found to be large, unstable and have low entrapment efficiency. ML is known to degrade upon exposure to air and light [4], and this restricts its application in food and nutraceutical products. Generally, it is considered that oxidization and crosslinking are two of the major mechanisms of photoreaction occurring in the unsaturated bonds, which promote the formation of pores in the lipid bilayers and the concomitant membrane leakage. It was observed that the hydrocarbon chains, especially the unsaturated chains, are more prone to lipid oxidation in the liposomes that are made of mixed lipids [5]. Lipid oxidation is a radical chemical reaction which causes the cleavage of hydrocarbon chains, or in the presence of adjoining double bonds, leads to the formation of cyclic peroxides [6]. The initial step, which includes the abstraction of the hydrogen atom from a lipid chain, takes place due to the exposure to light or low concentrations of metal ion contaminants. The lipids which are very susceptible to this step include those with a double bond, as the unsaturation permits the delocalization of the rest of the unpaired electrons, which further decreases the energy in this state. Hence, polyunsaturated lipids are more prone to oxidative degradation. Presence of oxygen in this step leads to the cleavage of the hydrocarbon chain and the formation of peroxides. The mechanism responsible for this release is based on membrane permeability that results from the oxidation of unsaturated lipids. Membrane permeability is due to the pores formed in the lipid bilayer, which leads to the leakage of chemicals from the nanoparticles. In addition to this, alteration in the rigidity and conformation also occurred, thereby leading to decreased liposome shelf-lives. Thus, to improve the stability of these ML-based liposomes, membrane additives can be added to the formulation. Cholesterol (CH) has traditionally been included to decrease the permeability and strengthen the lipid membrane. Additionally, non-ionic surfactants with a high hydrophilic-lipophilic balance (HLB), such as Tween80 (TW80), cannot form vesicles without CH due to their large hydrophilic head groups [7]. Squalene (SQ, C_30_H_50_), a natural isoprenoid compound, can be exploited because it possesses anticancer and antioxidant properties [8,9], inhibits the development of various tumors [10,11], is well-tolerated intravenously or orally [9] and has wide applications in the food and biomedical industries. According to Richens et al. [12], oily natural substances such as SQ can modify the membrane dipole potential. However, there is insufficient exploration of SQ in liposomal formulations. Thus, in this study, SQ was used together with ML to form mixed liposomes. Furthermore, the addition of different concentrations of non-ionic surfactants was found to affect the hydrophilicity, particle size, fluidity and integrity of mixed liposomes [13]. It was suggested that elastic liposomes could respond to external stress by rapid shape transformations [14]. The hypothesis of surfactant integration with liposomes is also supported by another study [15].

Co-extrusion refers to a technique that forms vesicles wherein the molecules (i.e., core) are surrounded by a physical barrier (or shell) [16]. This is the microencapsulation technique used for preserving the oil by creating a shell wall barrier for enclosing the oil core, which was further fortified using an antioxidant [17,18]. This encapsulation technique reduces undesirable taste like the bitterness of the core chemicals as it decreases the contact of the core with the oral taste receptors. As such, the derived antioxidant-fortified oil microbeads can be used as a food ingredient or directly consumed as nutritional supplements. The shell wall matrix influences the shape, size and integrity of the microbeads and also affects the stability of the core substances and encapsulation efficiency [17]. Batzri and Korn [19] developed the extrusion method, which has many advantages as compared to other methods that are commonly used for preparing liposomes. It is a one-step, simple, inexpensive, rapid and reproducible method, which avoids the use of harmful solvents and stronger forces that could disrupt the liposomes and cause a leakage of the encapsulated substances. Moreover, according to the literature, this technique does not cause any oxidative changes or degradation of the lipid mixture or the encapsulated chemical molecules [19,20].

Therefore, the use of various materials for the production of liposomes via an extrusion technology is expected to confer triple benefits: enhanced formation of stable liposomes for effective nutraceutical/food-grade delivery applications, increased nutritional value with the addition of naturally occurring SQ and reduced photodegradation rate via the use of appropriate ratios of liposome ingredients. To date, no study has investigated the development of an optimal liposomal formulation using a combination of crude soybean ML, CH and TW80 to exploit its potential as a novel food-grade nutrient delivery system for functional lipid SQ. In this study, an extrusion technology was employed, as a simple low-energy method, to prepare the liposomes. The main aim of this study was to establish whether the application of ML, CH and TW80 under different combination ratios was effective in forming stable liposomes possessing good antioxidant capacity. Besides that, this study aimed to find the optimal formulation to produce SQ-loaded liposomes with the least degradation against UV light by using a full factorial design. XRD analysis was performed to determine the crystallinity of the liposome complex and to compare it to the free liposome.

## 2. Results and Discussion

### 2.1. Particle Size, PDI and Particle Size Change Rate of Different Liposome Compositions Exposed to UV Light

The liposomes composed as reported in Table 1 were produced via an extrusion method and had a mean particle size less than 200 nm (Table 2). The PDI of the studied vesicles had values from 0.125 ± 0.003 (high homogeneity) to 0.439 ± 0.002 (lower, but acceptable homogeneity, since PDI < 0.5), indicating homogenous populations of vesicles (Table 3). No significant difference (*p* > 0.05) was found between the sizes of the liposomes prepared with different compositions (Table 2). The presence of various concentrations of ML, SQ, CH, or TW80 did not affect the liposome particle size.

In an attempt to examine the physical stability of liposomes produced with different formulations, all 20 samples were irradiated by UV light (280–320 nm) at ambient temperature for 6 h. Table 3 lists the liposome particle percentage increase (%increase). Particle size showed consequential variations (Table 2), as it was increased due to exposure to UV light, suggesting the photochemical destruction of products due to absorption of photon energy. This is explained by a drastic change in the liposome bilayer conformation. UV irradiation alters the physical properties of phospholipid membranes by disturbing the order and lipid packing and therefore induces an increase in membrane fluidity and permeability [21]. Principally, photon energy emissions can aggravate membrane disorders if unsaturated and other lipids are present in liposomes, clearly due to the formation of free radicals through processes such as one-electron redox reactions, high-energy radiation and photolysis, and thermal homolysis of the bonds [22]. Such processes can also change the polarity of the dispersion and decrease the pH of the solution and thus the stability of lipid particles. We observed that the simultaneous interactions of ML and CH imposed a significant impact (*p* < 0.05) and that more liposomes that are sensitive to UV strength were formed. The interaction effects of ML and CH on size increase after UV exposure are shown in Table 2. As outlined by Freitas and Müller [23], another consequence of changing the system due to light exposure is a reduction of the zeta potential (ZP). Supported by a sufficient reduction of repulsive forces, particles can interact to form a gel network. Therefore, UV light breeds a more turbid suspension, which is more pronounced at a higher ML concentration. From another perspective, as a consequence of photodegradation, the CH present within the bilayers might unfold due to the predominant effect of UV light on the membrane damage resulting from highly disordered polyunsaturated fatty acids (PUFA) chains and exposed hydrophobic patches that could promote particle aggregation. Another reason could be the limited concentration of CH consumption towards ML. In this context, its condensing and ordering effects may be practically confined. Hence, although CH is known to modulate molecular organization, the liposome bilayers could only poorly resist disruption against UV light because they might not contain adequate CH as a space-filler needed for the formation of a close-packed lamellar system. Its unequal affinity for different lipids provokes the formation of domains [24]. PUFA chains with their multitude of rapidly changing conformations push away the rigid steroid moiety so that PUFA-containing phospholipids have poor affinity for CH. Similar findings were reported by Wassall and Stillwell [25].

**Figure 1:**
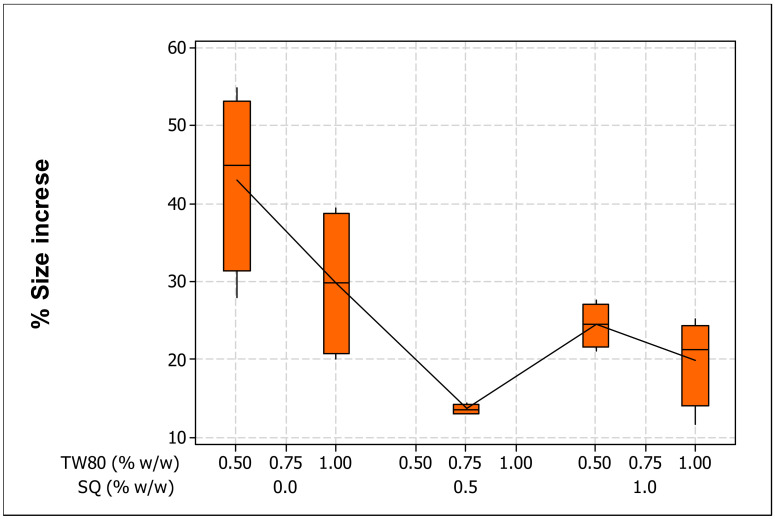
Size increase (%) of the prepared liposomes after UV exposure (280–320 nm, 6 h) affected by the effect of squalene (SQ) and Tween80 (TW80) concentrations.

The SQ-liposomes remained less degraded, with this result confirming that the addition of SQ fractions into the ML-based liposomes made them crucially stable to UV light (Figure 1). As reported by Bhalekar et al. [26], the energy imparted by the light excites vibrational motions in molecules inside the complex, so changes in both lengths and angles of bonds occur, and electronic transitions and cleavage of chemical bonds may occur. Introducing SQ might affect vesicular integrity and avoid further liposome disintegration. There are several possible explanations for this outcome. Firstly, the “fit” between the two imperfect adjoining chains could adhere well after bringing SQ into lipid bilayers containing mono- and poly-unsaturated lipid chains. This can be confirmed by the TEM results and the earlier mentioned hypotheses [27], which stated that SQ is oriented parallel to the plane of the bilayer and at its center, i.e., sandwiched between the two monolayers, and this might generate a stabilization of the membrane structure and broad label distribution. Moreover, the flexibility of the SQ molecule may help to partially extend into the lipid acyl chains and increase the acyl chain dynamics, preventing them from extra damage. Secondly, it is assumed that the binding of long linear hydrophobic lipid (SQ) to acyl chains of phospholipids makes a more stable lipidic complex as compared to SQ-free phospholipid bilayers. Basically, aliphatic organic molecules have stronger interactions than aromatic compounds, because branches on a carbon chain will reduce the hydrophobic effect of that molecule, and a linear carbon chain can produce the largest hydrophobic interaction, producing steric hindrance by carbon branches. This dynamic is consistent with the findings of Ott et al. [28], who used SQ for dual delivery of hydrophilic and lipophilic actives. They stated that the steric impediments of SQ could provide delayed release functionality for nanostructured lipid carriers.

It is thought that a single-chain surfactant makes liposomes flexible, as they have better hydration and rheology, which are responsible for their elasticity [15]. During the extrusion process, elastic liposomes self-modify their form and pass through the pore much smaller than their own diameter. Shape variation is only possible because the surfactant moves all the way through the bilayer into the zones of major tension [29]. As displayed in Table 4, the increased use of TW80 appeared to have a significant (*p* < 0.05) effect that improved the resistance of liposomes against UV light, in terms of their increase in size. This could be due to the electric repulsion of droplets in the dispersion, which causes the formation of an integral film together with a higher amount of ML at the membrane interface, resulting in higher resistance towards coalescence, or allowing a short-term stabilization of the new interface until the ML gradually becomes available during UV exposure. As stated previously by Tai et al. [13], when surfactants attach to liposome, they can benefit the liposomes via emulsification and modification mechanisms. Emulsification refers to the stabilization of the liposomal vesicles in the aqueous phase, and modification elucidates the transformation of the physical and chemical structures of the membranes to provide steric hindrance to decrease phospholipid hydrolysis. Equally, non-ionic surfactants such as TW80 induce droplet break-up during the homogenization process [30] and stabilize emulsions by adsorbing rapidly onto the droplet surface, such that the surfactant forms a screening layer around the droplets, decreases the interfacial tension between the organic phase and aqueous phase, and thus, reduces the strength of droplet interactions. Ultimately, the droplet avoids coalescence as a consequence of the chemical degradation of the phospholipids in the process of UV irradiation. The appropriate use of TW80 (not less than 0.5%, *w*/*w*) increased the flexibility and reduced surface tension and the radius of the liposomes, which led to the maintenance of phospholipids in small vesicles even when exposed to UV light. However, upon the increase of TW80 concentration, a relative decrease in size stability was observed. This decrease may be due to the more flexible liposomes breaking upon UV light irradiation, resulting in SQ leakage from the liposomes that subsequently led to an abrupt increase in the average particle size.

### 2.2. Zeta Potential (ZP) of the ML-Based Liposomes

All liposomes prepared in this study displayed a negative charge ZP, varying from −17.35 to −26.40 mV (Table 3). The ZP significantly (*p* < 0.05) became less negative as the concentration of ML incorporated in the liposome formulation was increased. Primarily, the negative ZP of the ML-based liposomes was due to the various anionic phospholipid fractions. The improvement of the ZP values at 5% (*w*/*w*) ML concentration may be explained by the decrease in the heterogeneity of the system, which was supported by the improvement in both the original size and %increase of size values (see Table 2 and Table 3).

Surface charge was also positively influenced (*p* < 0.05) by the gradual increase of CH (Table 4). A higher ZP absolute value might contribute to the enhanced stability of ML-based liposomes by CH. In terms of CH addition, it is hypothesized that the ZP would be related to the anomalous type of hydrogen bond between the methyl hydrogen of the choline group and the hydroxyl oxygen atom of CH. The strength of the C‒H⋯O interactions promoted the presence of an electronegative atom next to the donor C-H group [31]. Our results are in agreement with Liu et al. [32], who demonstrated that the incorporation of CH into the egg-PC vesicles can elevate the ZP. They showed that the incorporation of CH into the bilayer causes lipid vesicles to change their packaging geometrical structures, which include lipid vesicle size, the curvatures of the surface bilayer and the surface bilayer rigidity.

Table 4 indicates that the ZP of the prepared liposomes was significantly (*p* < 0.05) affected by the presence of SQ, as it became more negative as the concentration of SQ increased. SQ-liposomes may possess acceptable kinetic stability due to an increasing number of strong hydrophobic interactions (as outlined previously). The presence of SQ and the functional lipids in the membrane led to an increase in the steric hindrance and electrostatic repulsion of the liposomes. However, further increase of SQ caused the ZP to slightly decrease.

The ZP values varied considerably (*p* < 0.05) in a negative manner relative to TW80 concentration (see Table 4). The presence of TW80 at extended amounts could disturb the charge of ML at the surface of vesicles. Tasi et al. [33] proved that the incorporation of some Tween surfactants into the PC liposomal bilayer could affect the ZP, and this result may be attributed to the bulkhead group (CH_2_–CH_2_–O)*_n_* of Tween surfactants. The strongest negative ZP was observed for the formulation with the highest ML to TW80 mass ratio (6:1). This implied that, in contrast to ionic surfactants, non-ionic surfactants, such as TW80, have only a small contribution to the total surface charge. In fact, the use of surfactant beyond a concentration that is necessary for saturating the internal part of the double layer may result in the reduction of the ZP as a result of expanding the diffuse layer. Furthermore, due to the high concentration, excessive coverage at the interface of the liposome by the surfactant will cause an increase in the width of the diffuse layer and hence a decrease in ZP. According to Table 3, F8 and F11 showed the least stability in terms of ZP (−19.31 and −17.35 mV, respectively) among the formulations studied, and both samples were prepared using the maximum amount of surfactant. Thus, it is suggested that the medium (center point) concentration of non-ionic surfactant may act favorably at both stages of surface coverage.

### 2.3. Assessment of TEAC, and the Percentage Loss of TEAC against UV Light

In order to assess the antioxidant capacity of the liposomes prepared with different compositions, their DPPH scavenging activity was measured and compared with that of a synthetic antioxidant trolox. The TEAC values significantly (*p* < 0.05) improved following the use of greater amounts of ML, SQ and CH in the formulation (Table 4). However, the addition of TW80 appeared to have no significant impact (*p* > 0.05) on the TEAC values. As proposed by Pérez-Rosés et al. [34], TW80 is inactive against the stable free radical DPPH•, signifying that it has a very limited ability to scavenge free radicals. Still, TW80 could yield a positive coefficient (Table 4) for TEAC values in the produced liposomes. As mentioned earlier, TW80 significantly reduced the interfacial tension of the vesicles, which in turn promoted the formation of smaller vesicles in a larger volume. As such, the capability of the liposomal systems to scavenge more free radicals improved.

**Figure 2:**
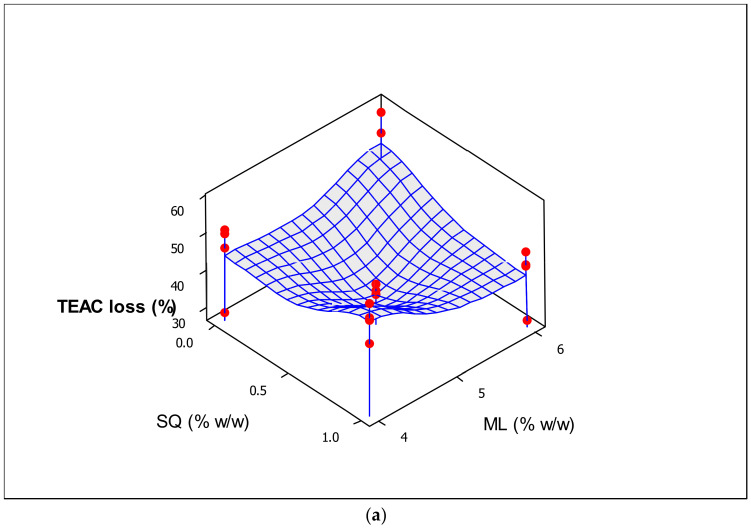

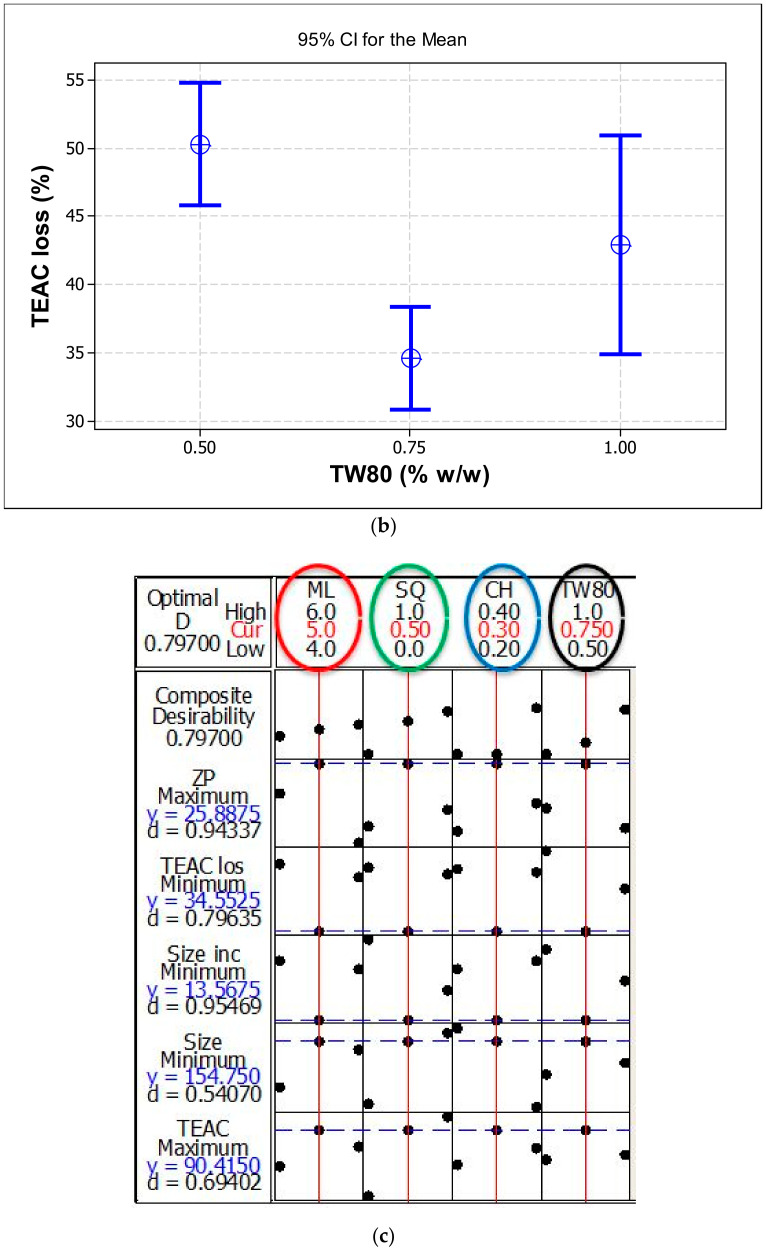
Trolox equivalent antioxidant capacity (TEAC) loss (%) of the prepared liposomes against UV light (280–320 nm, 6 h) affected by the (**a**) combined effects of mixed soy lecithin (ML) and squalene (SQ) concentrations and (**b**) effect of Tween80 (TW80) concentration, and (**c**) multiple response optimizer showing the optimum concentrations of (5% (*w*/*w*) mixed soy lecithin (ML), 0.5% (*w*/*w*) squalene (SQ), 0.3% (*w*/*w*) cholesterol (CH) and 0.75% (*w*/*w*) Tween80 (TW80)) in order to achieve the maximum zeta potential (ZP) and trolox equivalent antioxidant capacity (TEAC), minimum particle size and loss of TEAC, and minimum increase in size.

For the purpose of evaluating the antioxidant stability of the liposomes in the presence of various composition concentrations, the oxidant inhibitory activity of all samples was measured via DPPH assay before and after UV irradiation for 6 h and standardized via TEAC assay using the synthetic antioxidant trolox (Table 2). Unlike the results reported in Section 3.1 on changes in particle size, higher concentration of natural ML provided better protection to the liposomes against degradation because ML is a good antioxidant and prevents the penetration of peroxyl radicals [35]. Generally, when exposed to UV light, the TEAC decreased considerably. However, the decrease in TEAC became non-significant with increasing amounts of ML, CH and SQ. This was most probably due to the increase in membrane thickness following the addition of ML as well as the attachment of a higher amount of SQ to the acyl chains of the phospholipids (Figure 2a). The attachment efficiency of SQ could be influenced by its lipophilic character and its tendency to merge with the ML membrane bilayer. The research established by Gruner [36] demonstrated that the constraints of hydrocarbon chain packing, by virtue of the anisotropic packing requirements in the hexagonal phase tube, are reduced by filling the interstitial spaces between the hexagonally packed phospholipid tubes with the hydrophobic molecules. As outlined previously by Lohner et al. [37], SQ tends to dissolve in the most disordered region of the hydrophobic core, which enables these molecules to fill the perimeters and corner regions of the hexagons. Consequently, an ordered concentrated bilayer can block the UV light from penetrating into the interior of liposomes, thereby leading to less SQ degradation and TEAC loss.

As shown in Table 4, a significant (*p* < 0.05) positive impact on the percentage loss of TEAC was observed for TW80 (also see Figure 2b). These results are consistent with the findings on the %size increase (shown in Table 3). Liposomes prepared in this study were composed of a mobile hydrophilic surfactant working as edge-activators and inserted into a biocompatible substrate of ML phospholipids. The important difference between surfactant-integrated liposomes and surfactant-free liposomes is the high and stress-dependent adaptability of such surfactant conjugated vesicles. This difference is demonstrated in the driving force provided by the osmotic gradient between the outer and inner layers of the liposomes. Edge activators with a high radius of curvature could obviously increase the fluidity and flexibility and enhance the deformability of the bilayers [38]. It appears that such flexible membranes are suitable for maintaining the integrity of the liposomal vesicles, engendering effective durability of the free radical scavenging system against UV-light-induced degradation. This may be because TW80 could sterically shield the lytic effect of phospholipids in the interface. From another standpoint, when exposed to UV light, phospholipids may be readily exchanged with the environment. TW80 is viscous and may minimize the exchange of phospholipids from the interfacial region. Moreover, the higher amount of TW80 could help in the wetting and dispersion of the very hydrophobic SQ molecules and enhance its solubility [39]. Thus, DPPH could easily capture H^+^ atoms from the SQ in such systems that are exposed to UV light.

Taken together, our results suggest that the center point runs, with interacting surfactant and lipids of intermediate concentrations, could generate dominant stabilization in the liposome membrane structure. Hence, the center point composition was chosen as the optimal point (Figure 2c and Table 5) in accordance with the resulting liposome’s high stability against UV.

### 2.4. Structural Analysis by FTIR Spectroscopy

**Figure 3:**
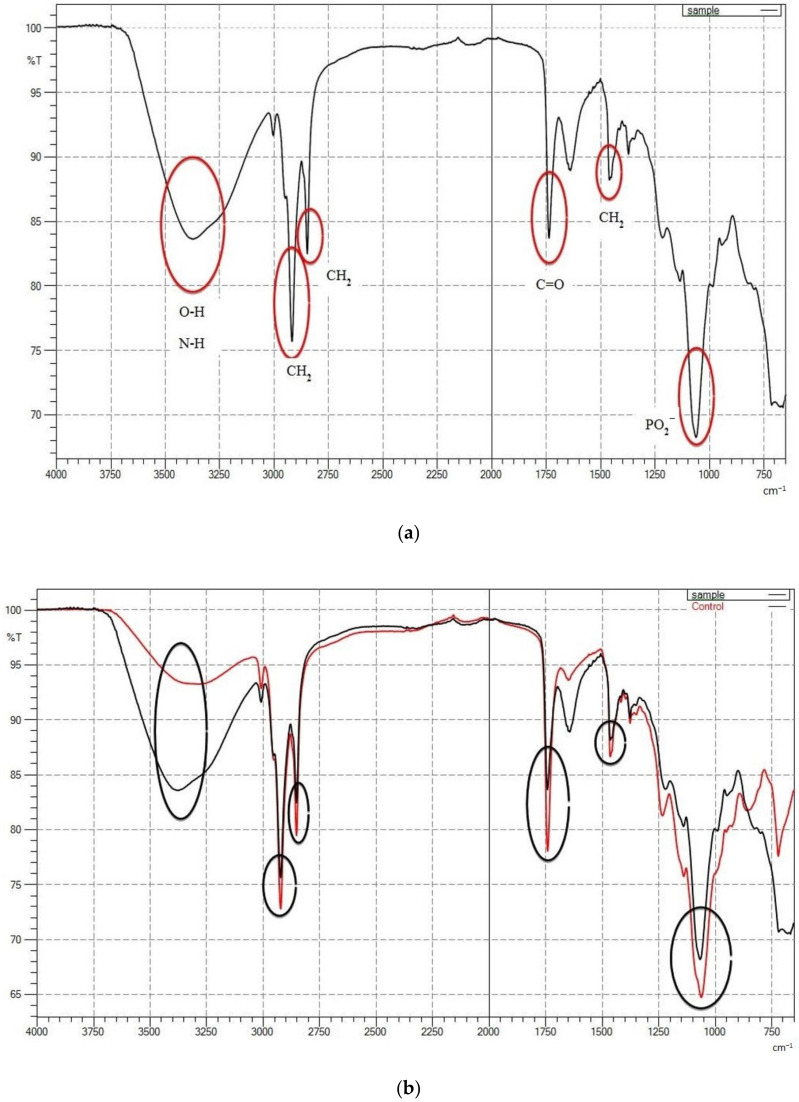

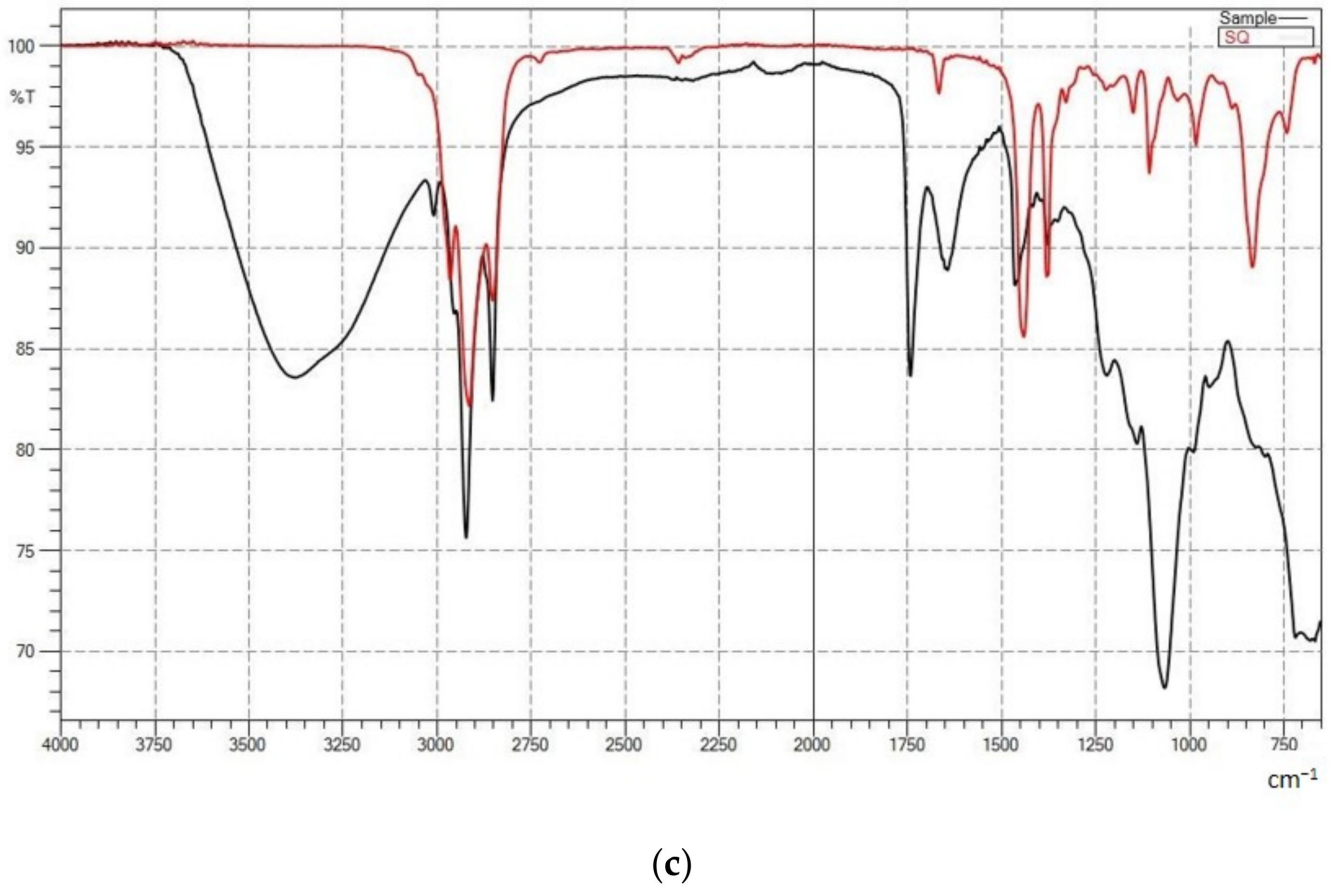
FTIR spectra showing (**a**) SQ-loaded liposome (5% (*w*/*w*) mixed soy lecithin (ML), 0.5% (*w*/*w*) squalene (SQ), 0.3% (*w*/*w*) cholesterol (CH) and 0.75% (*w*/*w*) Tween80 (TW80)), (**b**) overlap spectra of squalene (SQ)-loaded (black) and squalene (SQ)-free liposomes (5% (*w*/*w*) mixed soy lecithin (ML), 0.3% (*w*/*w*) cholesterol (CH) and 0.75% (*w*/*w*) Tween80 (TW80)) (red) and (**c**) overlap spectra of pure SQ (red) and SQ-loaded liposome (black).

FTIR spectra were collected to confirm the presence of SQ in ML-based liposomes. The following spectral regions were revealed: amino +NH, hydroxyl–OH asymmetric stretching vibration (1620–1660 cm^−1^), phosphate asymmetric stretching vibration PO_2_^−^ (1220–1260 cm^−1^), carbonyl stretching mode C=O (1680–1800 cm^−1^) and symmetric CH_2_ stretching vibration (2850–2855 cm^−1^) (Figure 3a). The pure SQ exhibited a characteristic peak at 2920 cm^−1^ due to CH_3_ stretching [40]. The characteristic peaks appeared in the FTIR of SQ-embedded liposome, which confirms the presence of SQ in the optimized sample (Figure 3b). High overlap (seen in Figure 3b) with methylene stretching mode at 2921 cm^−1^ is important because the intensity of these bands is used to measure the amount of SQ adsorbed on the liposome bilayers. It proved a remarkable decrease in the liberational freedom of the chains in the center of the bilayer. A possible explanation for this ordering and stabilizing effect is the hydrophobic interaction between the SQ molecule and the acyl chains of ML, which results in a greater ordering of the lipid bilayer structure without leading to a loss of the cooperative transition. At a favorable concentration (0.5%, *w*/*w*), SQ molecules are unable to disturb the tight packing of the lipid acyl chains. Hence, the cooperative transition is still observed. The molecule is mainly localized in the cooperative region of the bilayer and interacts with the methylene and terminal methyl groups, causing significant changes in the physical parameters of the membrane.

According to Figure 3c, the bands in the phosphate head group region shifted towards higher transmittance (%T) upon incorporation of SQ into the membranes. This effect can be readily interpreted in terms of hydrogen bonding to the PO2– group, either SQ or water molecules, influenced by the presence of the SQ addition in the system. A shift to higher transmittance along with a broadening of this band is indicative of a higher degree of hydration of the head group. Moreover, the addition of SQ into the ML membrane system caused a narrowing in the bandwidth, which may indicate the immobilization of the phosphate groups.

### 2.5. XRD Analysis

**Figure 4:**
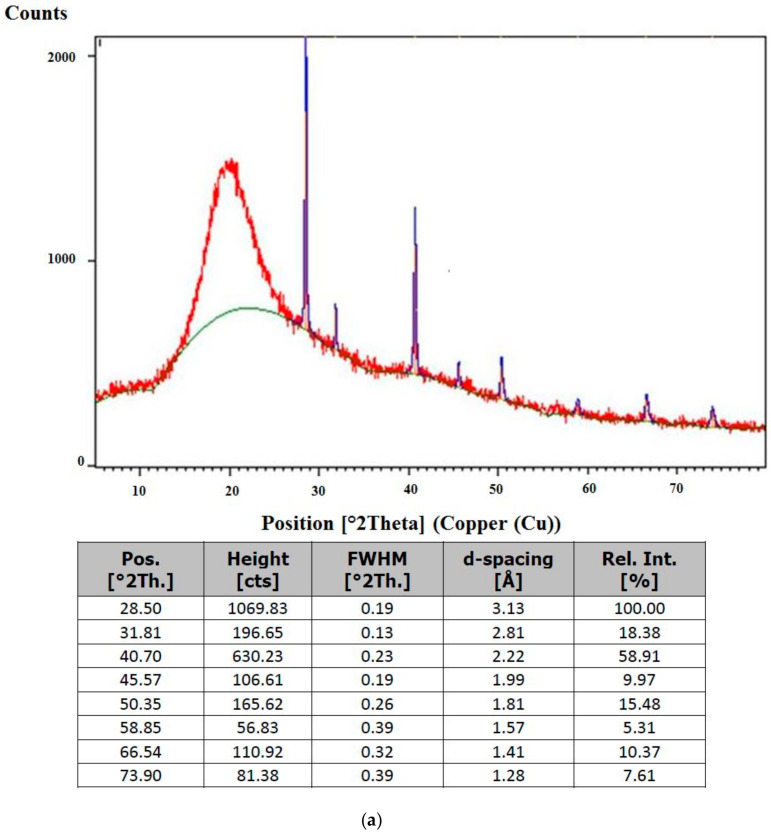

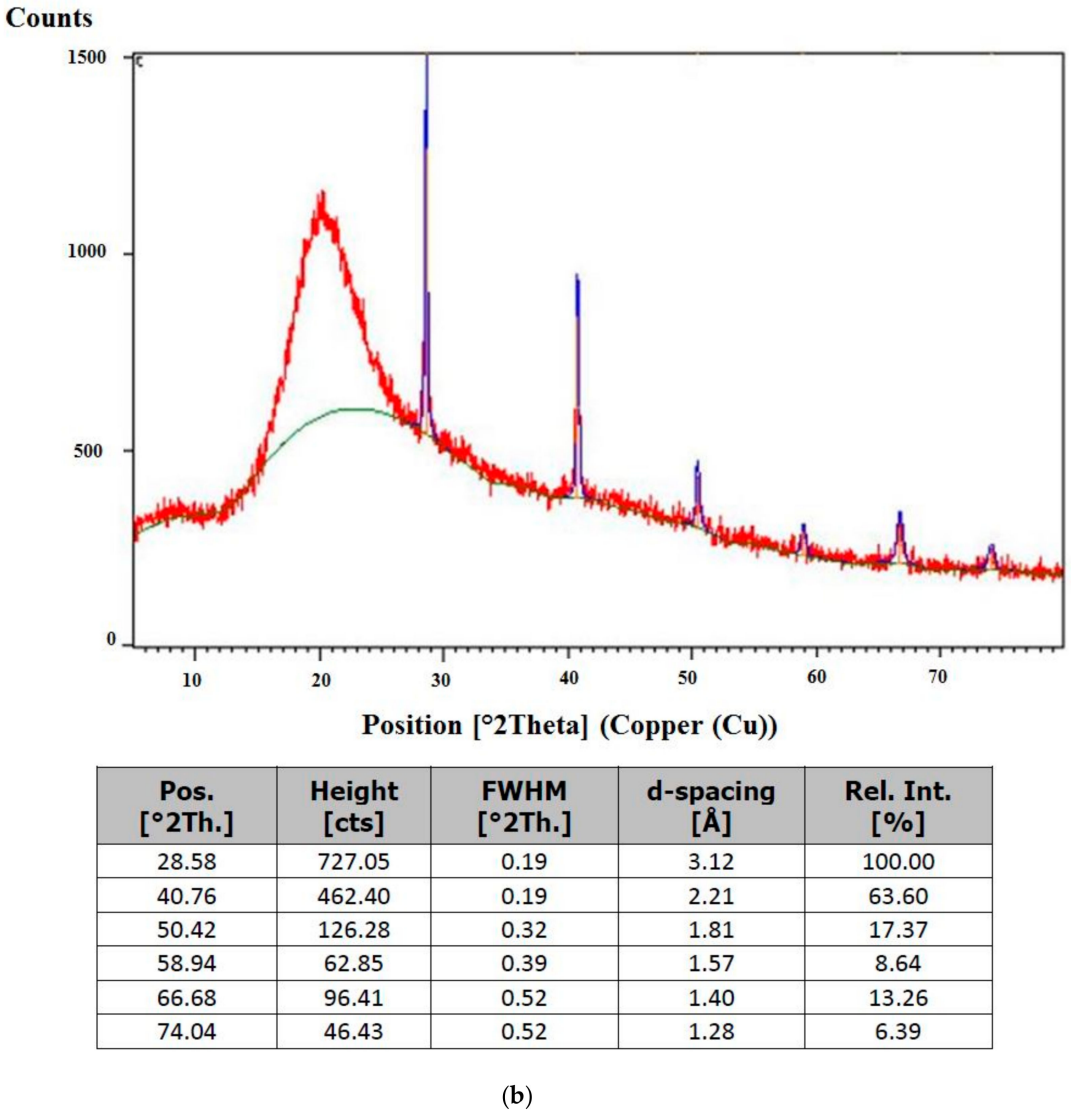
XRD ((**a**,**b**) show the diffractogram and peak list of squalene-loaded liposome (5% (*w*/*w*) mixed soy lecithin (ML), 0.5% (*w*/*w*) squalene (SQ), 0.3% (*w*/*w*) cholesterol (CH) and 0.75% (*w*/*w*) Tween80 (TW80)) and squalene (SQ)-free liposome (control) (5% (*w*/*w*) mixed soy lecithin (ML), 0.3% (*w*/*w*) cholesterol (CH) and 0.75% (*w*/*w*) Tween80 (TW80)), respectively).

The XRD patterns of the optimal SQ-liposome and its control counterpart (without SQ) are presented in Figure 4a,b, respectively. Basically, the peak position, peak width and peak intensity are considered as significant peak shape attributes in XRD. Accordingly, peak lists of the liposome samples are illustrated in Figure 4a, b to identify these factors accurately. The positions of diffraction peaks and the d-spacings that they represent provide information about the location of the lattice planes in the crystal structure. Each peak measures a d-spacing that represents a family of lattice planes. Each peak also has an intensity which differs from other peaks in the pattern and reflects the relative strength of the diffraction. In a diffraction pattern, the strongest peak is, by convention, assigned an intensity value of 100, and the other peaks are scaled relative to that value. Variations in the measured intensity are chiefly related to variations in the scattering intensity of the components of the crystal structure and their arrangement in the lattice. Some of the most dramatic variations are related to interference between the diffractions produced within the lattice, and these diffractions can produce systematic extinctions or greatly reduced intensities of peaks from certain lattice planes. Peaks with such a broad hump (a high full width at half maximum (FWHM) or a decrease of peak height) have a well-known confirmed characteristic phase which is amorphous or poorly crystalline in nature [41]. In addition, according to Li et al. [42], an amorphous precipitate presents a halo in the diffractogram without defined peaks due to the disordered arrangement of drug molecules in an amorphous form.

XRD results showed that the liposome samples had relatively uniform lattice distortion peaks, indicating an ordered lipid lattice (symmetric) and low surface tension of the nanoparticles. When compared with the control, higher (see height values) and narrower (see FWHM values) peaks existed in the spectrums of optimal SQ-liposome, indicating that the stereochemistry of SQ chain affected the crystallinity of the liposomal system, as it naturally had a more crystalline phase. The broader peak of the control diffraction pattern may be because of the combination of stresses present and smaller crystallite size than the optimal SQ-liposome composition. These XRD data supported the idea of occurring changes in the physical structure of the SQ-liposome after the addition of SQ, possibly in a more amorphous state (broadened nature of peaks). Similar observation has been made in other study, whereby the altered peak in the X-ray diffractograms of coriander essential oil-loaded chitosan microcapsules was attributed to partial transformation of crystal structure into an amorphous one due to encapsulation [43].

### 2.6. Stability of SQ-Loaded and Free Liposomes during Storage

To assess the long-term physical stability of the optimized SQ-loaded and free (control) particles (see Table 6) that were stored at 4 °C in the dark, changes in the particle size and ZP were observed over a period of 8 weeks. Overall, the physical stability of the optimized SQ-loaded formulation decreased less substantially in comparison with the control sample. There were no significant differences in the particle diameter as well as ZP values among the liposomes during the 4-week storage. The overall stability of the liposomes during 8 weeks of storage time suggested that there might be less aggregation of the liposomes due to high surface hydrophilicity of the nonionic surfactant (TW80) in the liposomes, which gave a more stable detergent film and kept the liposomes apart during 8 weeks of storage. In addition, a decrease in phospholipid hydrolysis and resultant reduction in the chemical breakdown of the liposomes formed with the cured ML was another advantage conferred by TW80, which helped the lipid vesicles in maintaining a stable particle size throughout the storage period.

The control sample slightly (*p* > 0.05) increased in size from below 168 nm to a maximum of 172.26 nm over a 4-week period (Table 6). The optimized SQ-loaded liposome exhibited a high stability during 4 weeks with averaged diameter of 160 ± 2.45 nm. In addition, the liposomal formulations showed no changes in ZP at the end of a 4-week storage period (Table 6). These results suggested that unlike the unloaded control sample, the optimized SQ-loaded particles showed better stability towards droplet aggregation and the increase in the particle size of the control may be explained by the liposome swelling and increased membrane fluidity in the absence of the membrane-stabilizing agent. The optimized SQ-loaded liposome displayed a relatively constant particle size and ZP, which can be attributed to the lower interfacial tension in the presence of SQ, resulting in the better storage stability until week 4, as compared to the control without SQ. Furthermore, the polymorphic transition can also be accelerated by the presence of a substance in the carrier [44]. This phenomenon is attributed to high interactions between lipids and the substance, which lead to a higher stability in comparison to nonleaded particles (see the XRD results).

It was interesting to find that for the optimized SQ-loaded liposome, the addition of SQ led to lower stability as compared to the SQ-free liposome (control), such that the particle size significantly (*p* < 0.05) increased from 167.66 to 175 nm in diameter, starting from week 6 until the end of the storage period, indicating that the SQ-containing liposome was unfit for storage of more than 8 weeks. The substantial size increase displayed by the optimized SQ-loaded liposome might be due to leakage of SQ from the liposome and thus, the system became more polydisperse because as a non-polar compound, SQ might easily permeate through the bilayer membrane. Our findings indicate that at 6 weeks of storage, only a slight increase (*p* > 0.05) was observed in the ZP values of all three nanoliposomes (Table 6). Usually, particle aggregation is less likely to occur for charged particles with pronounced ZP (>|20 mV|) due to the electrostatic repulsion between particles with the same electrical charge [45]. In addition, ZP remains practically unchanged in the absence of light exposure and at low temperatures. Accordingly, for all three liposomal formulations having ZP of more than −20 mV, which were stored at 4 °C in the dark (Table 6), particle aggregation was less likely to occur for charged particles (high ZP) due to the electrostatic repulsion. These results support the hypothesis that for suspension with a sufficient ZP, it is more likely that it remains stable for a certain period of time because the charged particles repel one another and thus overcome the natural tendency to aggregate over time.

For the optimized SQ-loaded liposome, the particle size and ZP again increased significantly (*p* < 0.05) after 6 to 8 weeks of storage (Table 6). Less pronounced suspension destabilization was observed for both the control particles. It is suggested that after 8 weeks of storage, no SQ adhered to liposome membrane because SQ lacks a hydroxyl group, which may anchor it to the interface, as this would have induced an increased aggregation. The optimized SQ-loaded sample showed a layer of the oil on the top of the suspension that can be considered as SQ that is released from the liposomes. This may be attributed to the effect of the storage time and low temperature and subsequent aging on the gel-to-liquid transition of the bilayers, together with possible chemical degradation of the unsaturated phospholipids, leading to defects in the membrane packing, as stated by Frenzel and Steffen-Heins [46] and Pawlikowska-Pawlęga et al. [47].

For the purpose of evaluating the effect of nanoencapsulation on DPPH scavenging ability, the antioxidant activity of the liposomes was measured via DPPH assay every week for up to 4 weeks at 45 °C, and was completed via TEAC assay using the synthetic antioxidant trolox (Table 6). The results revealed a descending trend in the TEAC values until the end of the storage period.

As expected, the TEAC loss of the control sample increased significantly (*p* < 0.05) during the first week of storage. The degradation rate increased sharply for the optimized SQ-loaded liposome starting from week 2. It is conceivable that after two weeks of incubation at 45 °C, a decrease in solubility of the lipids might occur and thus, particle growth and gelling could accelerate, resulting in a decrease in antioxidant activity of the nanosystems. These results agree with those of particle size and ZP that remained unstable after a 6-week storage at 4 °C in the dark, proving that fusion and breakage of the liposomes upon storage also posed a more important problem of electron-donating ability (DPPH), which may have resulted in the observed decreased TEAC. In addition, the degradation reaction of unsaturated lipids was conducted by maintaining them at 45 °C for 2 weeks.

These values continued to decrease with increasing storage time to 3 weeks for the studied samples, which appeared in the following order: optimized SQ-loaded sample < control. Nevertheless, in the optimized SQ-loaded liposome, the lower viscosity of the dispersed liquid phase caused a gradual decrease in SQ diffusion out of the lipid phase, resulting in a pronounced reduction of antioxidant activity of the system.

After 4 weeks, the control and optimized SQ-loaded samples experienced a significant decrease (*p* < 0.05) in the TEAC values from 46.86 and 93.09 µM to 31.04 and 79.96 µM, respectively. The substantial loss in the TEAC values of the samples might be explained by the degradation of the ML after fatty acids formation. As a consequence of free-radical-forming, higher free radicals were accumulated during the oxidative stress period and intensified the activities of the chemical interactions to destabilize the lipid vesicles.

Overall, these results suggested that DPPH scavenging efficacy of the liposome became lower after the incubation period for 30 days at 45 °C due to the gradual transformation of the soy ML through an aqueous phase, promoting the degradation of ML components via oxidation, hydrolysis, collision and merging.

### 2.7. Morphological Evaluation by TEM

**Figure 5:**
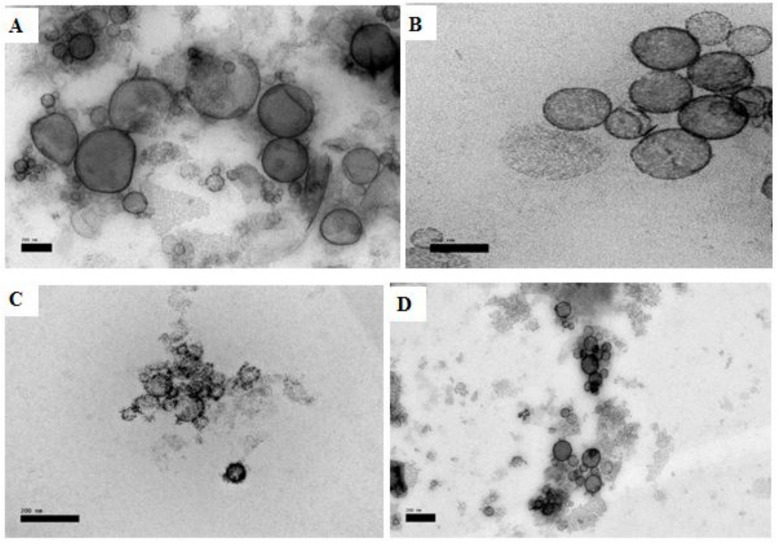
TEM micrographs of liposomes (scale bar: 200 nm): (**A**) control of optimized formulation (5% (*w*/*w*) mixed soy lecithin (ML), 0.3% (*w*/*w*) cholesterol (CH) and 0.75% (*w*/*w*) Tween80 (TW80)), (**B**) optimized squalene (SQ)-loaded liposomes (5% (*w*/*w*) mixed soy lecithin (ML), 0.5% (*w*/*w*) squalene (SQ), 0.3% (*w*/*w*) cholesterol (CH) and 0.75% (*w*/*w*) Tween80 (TW80)), (**C**) control of optimized formulation exposed to UV, (**D**) optimized squalene (SQ)-loaded liposomes exposed to UV light (280–320 nm, 6 h).

TEM images of optimized SQ-free and SQ-loaded liposomes are presented in Figure 5A,B, respectively. The TEM images of optimized liposomes revealed non-agglomerated liposomes with a mean particle size of 100–200 nm and were spherical in shape. In contrast, the SQ-free liposome was observed to be larger particles with less smoothness and some open-edge vesicles. Images of UV-treated liposomes (Figure 5C,D) revealed aggregated vesicles and unfolded structures due to the damaging effect of photon energy on the membrane bilayers, which was much more notable in the control of optimized formulation (without SQ) in Figure 5C.

## 3. Materials and Methods

### 3.1. Materials

ML (for phospholipid components see Table 7) used in this study was sourced from Ncalai Tesque, INC., (Kyoto, Japan). CH (95%) was purchased from Acros. SQ (99% pure, for synthesis) was obtained from Merck Inc. (Darmstadt, Germany). Polyoxyethylene sorbitan monooleates (Tween80) was purchased from Merck Inc. (Darmstadt, Germany). All other chemicals and reagents, such as chloroform (CAS n. 67-66-3), ethanol (CAS n. 64-17-5) and methanol (CAS n. 67-56-1) (Sigma Aldrich, Milan, Italy), were of analytical grade. Deionized water was used throughout the study.

### 3.2. Experimental Design and Statistical Analysis

A 4-factor, 3-level full factorial design was selected to evaluate and optimize the formulation parameters. The independent variables studied were the mass ratios of ML (X_1_), SQ (X_2_), CH (X_3_) and TW80 (X_4_), while the dependent variables were liposomal particle size (Y_1_), particle size value change rate (Y_2_), polydispersity index (PDI) (Y_3_), zeta potential (ZP) (Y_4_), trolox equivalent antioxidant capacity (TEAC) (Y_5_) and percentage loss of TEAC (Y_6_). The statistical analysis of the factorial design formulations was performed using Minitab statistics software 16 (Minitab Inc., State College, PA, USA). Statistical significance was established at *p* < 0.05, as determined by Tukey’s test. The application of full factorial design allowed us to provide proper empirical equations for the prediction of the physicochemical properties of the liposomal systems as target responses. The center point was repeated three times, and experiments were randomized to minimize the effects of unexplained variability in the actual responses due to extraneous factors. Finally, the optimization was carried out to find the most desirable formulation condition, resulting in the optimal liposomal formulation. All values were the means ± standard deviation (SD) of two independent experiments and all measurements were performed in triplicate.

### 3.3. Liposome Preparation via the Extrusion Method

The mass ratios of materials were selected based on our preliminary study and those reported by other studies [40,48]. Liposomes were prepared by varying the proportions of ML, CH, TW80 and SQ. The extrusion method was used to form nano-sized lipid vesicles, with slight modifications [19,49]. Briefly, ML, CH, TW80 and SQ were mixed (see mass ratios in Table 1) and dissolved in a mixture of chloroform and ethanol (1:1). Solvent removal was performed using a rotary evaporator (Rotary Evaporator, Ultra Lab, New Delhi, India). The lipid film was then hydrated with phosphate buffered saline (PBS; pH 7.4, 0.05 M) by rotation (60 rpm, 1 h) above the lipid transition temperature (T_m_) (60 °C), followed by 1 min bath sonication at 60 °C to form uniform liposome suspensions (Loba Life, Mumbai, India). Large unilamellar vesicles (LUV) were prepared by extruding eight times through a 100 nm polycarbonate membrane (Millipore, Burlington, MA, USA) using an Avanti Mini-Extruder (Avanti Polar Lipids, Inc., Alabaster, AL, USA). The temperature during extrusion was carefully controlled and ensured to be above T_m_ of the vesicle membrane to minimize lipid loss during the extrusion process.

### 3.4. Particle Size, Polydispersity Index (PDI) and Particle Size Stability Measurement

The particle size (z-Ave) and PDI, which is an indicator of the width of particle size distribution, ranges from 0 (monodispersed) to 1 (very broad distribution) were measured using a dynamic light scattering instrument (Zetasizer Nano ZS, Malvern Instruments, Worcestershire, UK). All solutions were diluted 10-fold in PBS before measurement. Determinations of the liposome particle size after UV light exposure (280–320 nm) were performed to examine changes in the physical properties of the liposomes produced using different formulations. All measurements were carried out 24 h after liposome preparation and were conducted in triplicate.

The change in particle size was obtained by calculating the mean particle size of the treated and untreated liposomes under the same process described above. The particle size value change rate caused by UV light exposure given as a percentage was calculated using Equation (1):

(1) $$\mathrm{Value}\;\mathrm{change}\;\mathrm{rate}\left(\%\right)=\frac{\left[\mathrm{UV}\;\mathrm{treated}\;\mathrm{particle}\;\mathrm{size}\;\left(\mathrm{nm}\right)-\mathrm{Initial}\;\mathrm{particle}\;\mathrm{size}\;\left(\mathrm{nm}\right)\right]\times 100\;}{\mathrm{Initial}\;\mathrm{particle}\;\mathrm{size}\;\left(\mathrm{nm}\right)}$$

where the value change rate represents the percentage increase of the particle size of liposomes after UV light irradiation.

### 3.5. Zeta Potential (ZP)

The Zetasizer Nano ZS (Malvern Instruments Ltd., Worcestershire, UK) was used to determine the surface charge of the liposomal batches. The samples were diluted 1:10 in their original solvent. All measurements were carried out at 25 °C after 24 h of preparation. Each reported value was the mean of six readings from two replicates.

### 3.6. Antioxidant Activity and Antioxidant Activity Change Rate Assays

The antioxidant activity was evaluated via the DPPH method (DPPH = 2,2-diphenyl-1-picrylhydrazyl) [50]. Each sample solution (50 µL) was diluted to 10.0 mL with ethanol, followed by the addition of 2.5 mL of a DPPH solution in ethanol (100 µM) and then mixed under vortex agitation. After 30 min of reaction time at 25 °C in the dark, the resulting sample was analyzed spectrophotometrically (Agilent Technologies, Basel, Switzerland) at 517 nm using ethanol as a blank. The control mixture was prepared by replacing the sample with 50 µL of ethanol. The antioxidant activity of the liposome, given as a percentage (%antioxidant activity), was calculated by Equation (2):

(2) $$\%\mathrm{Antioxidant}\;\mathrm{activity}\;=\frac{\mathrm{Ac}-\mathrm{As}\times 100}{\mathrm{Ac}}$$

where, As and Ac represent the absorbance of the sample and the control, respectively.

The %antioxidant activity was expressed in trolox equivalents antioxidant capacity (*TEAC*). The *TEAC* was determined using Equation (3):

(3) $$TEAC\;\left(µM\right);\;y=0.4178x\;-\;5.9073$$

where *TEAC* corresponds to the trolox concentration (µM). Equation (3) was obtained from the plot of trolox versus %antioxidant activity (R^2^ = 0.9809, *n* = 5).

The loss/degradation of *TEAC* (%) as a result of photodegradation (280–320 nm, 6 h) was then obtained by using Equation (4):

(4) $$TEAC\;\mathrm{loss}/\mathrm{degradation}\;\mathrm{rate}\;\left(\%\right)\;=\frac{\left(TEACb\;–\;TEACa\right)\;\times \;100\;}{TEACb}$$

where *TEACb* and *TEACa* are the percentage of TEAC before and after UV light irradiation, respectively.

### 3.7. Structural Analysis of Liposomes by Fourier-Transform Infrared Spectroscopy (FTIR)

Infrared absorption spectra were reordered with a FTIR (FTIR-8400S, Shimadzu, Japan). The spectra of pure SQ and SQ-loaded liposomes were recorded in the scanning range of 750–4000 cm^−1^ at a resolution of one data point every 0.6 cm^−1^ using a clean crystal as the background. Moreover, vibrational bands from the interface region (ester carbonyl stretch approximately 1725 to 1740 cm^−1^) and the head group (antisymmetric stretch at 1260 to 1220 cm^−1^, the choline group approximately 1100 to 1000 cm^−1^ and the N–H stretching band at 1000 to 950 cm^−1^) were analyzed. All measurements were performed at 20 °C.

### 3.8. X-ray Diffraction (XRD) Analysis

XRD patterns were evaluated in order to assess the crystalline state of the lipophilic molecules in freeze-dried liposomes. The experiments were conducted using Cu K_ɑ_ radiation by an X-ray diffractometer (Panalytical X’Pert Pro, Philips, The Netherlands) equipped with a Lynx Eye detector. The freeze-dried samples were placed on a glass sample holder and the diffraction patterns were recorded in the range of 2*θ* from 10 to 60° with a step size of 1°/min and a slit width of 6.0 mm.

### 3.9. Storage Stability

To evaluate the effect of nanoencapsulation on storage stability, the optimized SQ-loaded and free (control) particles were stored in a refrigerator (4 °C) for 2 months (a 2-week interval), and at 45 °C for 1 month (a 1-week interval). Their storage stabilities were analyzed in terms of the physical properties including determination of mean liposome size and ZP when stored in the former and antioxidant activities in the latter.

### 3.10. Transmission Electron Microscopy (TEM)

SQ-loaded and free liposomes were analyzed via negative stain electron microscopy using a transmission electron microscope (Hitachi H-7100, Tokyo, Japan). A drop of each liposomal suspension was applied to a copper-coated carbon grid. The excess was drawn off using a filter paper. An aqueous solution of ammonium molybdate (1%, *w*/*v*) was used as a negative staining agent. After 2 min at room temperature, the excess solution was removed using a filter paper and then examined under the electron microscope.

## 4. Conclusions

This study indicated the importance of choosing the correct composition to obtain a qualitative functionalization of liposomes. All ingredients used in the present study were non-toxic and had generally recognized as safe (GRAS) status (SQ). The functional lipids and surfactant concentrations were determinant factors controlling the physical and chemical properties of produced liposomes. We confirmed that the addition of SQ to the ML from soybean was efficacious for the homogeneity of the liposome systems. In addition, this study revealed the possibility of producing a suitable soybean ML-based liposomal formulation despite the low applied pressure of the preparation method. The XRD patterns of the liposomes show neither large broadening of the diffraction line nor very low intensity, suggesting that lattice distortion, structural disorder as well as instrumental effects were limited in the liposomal systems.

Owing to this study, it would be possible to add ingredients such as CH and TW80 at low concentrations in order to improve their stability against UV light stress. This work provided further evidence that SQ protected unsaturated phospholipids and membrane structures against damage caused by UV light, indicating SQ’s antioxidant properties. Based on the results of this study, it can also be concluded that the liposomal formulation containing 5% (*w*/*w*) ML, 0.5% (*w*/*w*) SQ, 0.3% (*w*/*w*) CH and 0.75% (*w*/*w*) TW80 demonstrated the desired unilamellar structure and had the optimal physicochemical characteristics, which include a favorable ZP value of −26.84 ± 0.48 mV, and enhanced particle size (15.09% ± 3.76%) and TEAC (35.69% ± 0.72%) stabilities against UV light treatment (280–320 nm) for 6 h. Overall, the ML-based liposomes prepared using a blend of CH and TW80 had great potential to dissolve the highly hydrophobic compound SQ and displayed acceptable stability.

## Figures and Tables

**Table 1:** Full factorial design for formulation compositions of liposomes.

Formulation Code	Runs	Mixed Soybean Lecithin (X_1_, % *w*/*w*)	Squalene (X_2_, % *w*/*w*)	Cholesterol (X_3_, % *w*/*w*)	Tween 80 (X_4_, % *w*/*w*)	Phosphate Buffered Saline (mL)
F1	1	6	0	0.2	0.5	93.3
F2	2 *	5	0.5	0.3	0.75	93.45
F3	3	4	0	0.2	1	94.8
F4	4	4	1	0.2	0.5	94.3
F5	5	6	1	0.2	0.5	92.3
F6	6	4	1	0.2	1	93.8
F7	7 *	5	0.5	0.3	0.75	93.45
F8	8	6	1	0.2	1	91.8
F9	9	4	0	0.2	0.5	95.3
F10	10	4	1	0.4	0.5	94.1
F11	11	6	0	0.2	1	92.8
F12	12	6	1	0.4	0.5	92.1
F13	13 *	5	0.5	0.3	0.75	93.45
F14	14	6	0	0.4	1	92.6
F15	15 *	5	0.5	0.3	0.75	93.45
F16	16	6	0	0.4	0.5	93.1
F17	17	4	1	0.4	1	93.6
F18	18	4	0	0.4	0.5	95.1
F19	19	4	0	0.4	1	94.6
F20	20	6	1	0.4	1	91.6

* Center points.

**Table 2:** Changes in particle size and trolox equivalent antioxidant capacity (TEAC) of the prepared liposomes before and after exposure to UV light (280–320 nm) for 6 h.

Formulation Code	Z-Average (nm) ± SD		TEAC (µM)	
	Before	After	Before	After
F1	176 ± 1.41	225 ± 3.53	44.44	24.59
F2	154 ± 2.82	176 ± 9.89	90.99	58.72
F3	152 ± 2.12	212 ± 8.48	31.68	22.58
F4	147 ± 7.77	178 ± 7.07	77.30	33.64
F5	178 ± 8.48	219 ± 3.53	86.73	46.33
F6	158 ± 4.94	192 ± 5.65	94.51	44.60
F7	155 ± 9.89	175 ± 4.24	86.23	59.13
F8	171 ± 8.48	207 ± 2.12	107.15	61.14
F9	144 ± 3.53	213 ± 1.41	40.29	20.14
F10	138 ± 4.24	176 ± 9.89	104.23	50.13
F11	169 ± 9.89	208 ± 3.53	54.70	25.53
F12	166 ± 5.65	209 ± 6.36	116.31	65.88
F13	156 ± 6.36	177 ± 4.24	91.01	59.80
F14	167 ± 2.82	228 ± 2.12	63.13	34.62
F15	154 ± 4.24	175 ± 7.07	93.43	58.82
F16	170 ± 2.12	241 ± 2.12	56.31	23.28
F17	155 ± 4.24	173 ± 2.82	98.43	53.29
F18	135 ± 7.07	209 ± 2.12	43.57	21.39
F19	150 ± 2.82	180 ± 1.41	39.53	21.34
F20	163 ± 3.53	204 ± 4.24	105.11	75.26

**Table 3:** The polydispersity index (PDI), zeta potential (ZP), size increase (%) and trolox equivalent antioxidant capacity (TEAC) loss (%) of the prepared liposomes.

Formulation Code	PDI ± SD	ZP (mV) ± SD	Size Increase (%)	TEAC Loss (%)
F1	0.243 ± 0.004	−19.80 ± 0.50	27.84	44.66
F2	0.238 ± 0.003	−26.33 ± 3.54	14.28	35.46
F3	0.415 ± 0.002	−22.23 ± 2.05	39.47	28.72
F4	0.125 ± 0.003	−24.40 ± 4.11	21.08	56.48
F5	0.423 ± 0.004	−20.60 ± 0.25	23.03	46.58
F6	0.184 ± 0.001	−22.50 ± 1.27	21.51	52.80
F7	0.249 ± 0.002	−26.40 ± 1.36	12.90	31.42
F8	0.409 ± 0.002	−19.31 ± 1.73	21.05	42.93
F9	0.325 ± 0.004	−23.12 ± 0.08	47.91	50.01
F10	0.173 ± 0.003	−25.56 ± 1.08	27.53	51.90
F11	0.407 ± 0.004	−17.35 ± 1.49	23.07	53.32
F12	0.433 ± 0.003	−23.16 ± 0.48	25.90	43.35
F13	0.246 ± 0.003	−25.46 ± 0.20	13.46	34.29
F14	0.439 ± 0.002	−20.58 ± 2.07	36.52	45.16
F15	0.251 ± 0.002	−25.36 ± 1.37	13.63	37.04
F16	0.430 ± 0.002	−21.75 ± 0.21	41.76	58.65
F17	0.155 ± 0.002	−25.20 ± 1.88	11.61	45.86
F18	0.406 ± 0.007	−24.32 ± 0.26	54.81	50.90
F19	0.309 ± 0.005	−23.37 ± 0.65	20.00	46.01
F20	0.305 ± 0.004	−20.77 ± 1.77	25.15	28.39

**Table 4:** Analysis of variance for the experimental variables as main and interaction terms of each response variable and corresponding coefficients fitted for particle size (nm), zeta potential (ZP), trolox equivalent antioxidant capacity (TEAC), particle size increase (%) and trolox equivalent antioxidant capacity (TEAC) loss (%).

Responses Interaction Effects	Significant Level								Main Effects		
		X_1_	X_2_	X_3_	X_4_	X_1_X_2_	X_1_X_3_	X_1_X_4_	X_2_X_3_	X_2_X_4_	X_3_X_4_
Particle size (nm)	*P value*	0.382 ^c^	0.126 ^c^	0.091 ^c^	0.788 ^c^	0.987 ^c^	0.899 ^c^	0.253 ^c^	0.157 ^c^	0.943 ^c^	0.831 ^c^
	*F ratio*	0.85	2.91	3.70	0.08	0.00	0.02	1.51	2.43	0.01	0.05
ZP (mV)	*P value*	0.000 ^a^	0.004 ^b^	0.000 ^a^	0.001	0.910 ^c^	0.217 ^c^	0.191 ^c^	0.876 ^c^	0.836 ^c^	0.480 ^c^
	*F ratio*	149.33	16.06	47.24	25.89	0.01	1.79	2.04	0.03	0.05	0.55
TEAC (µM)	*P value*	0.003	0.000 ^a^	0.007 ^b^	0.345 ^c^	0.391 ^c^	0.821 ^c^	0.303 ^c^	0.312 ^c^	0.535 ^c^	0.065 ^c^
	*F ratio*	17.43	277.21	12.92	1.01	0.82	0.05	1.21	1.16	0.42	4.58
Particle size increase (%)	*P value*	0.370 ^c^	0.001 ^a^	0.400 ^c^	0.008	0.055 ^c^	0.040 ^b^	0.056 ^c^	0.599 ^c^	0.128 ^c^	0.076 ^c^
	*F ratio*	0.90	30.83	0.79	12.01	5.06	5.96	4.97	0.30	2.89	4.14
TEAC loss (%)	*P value*	0.428 ^c^	0.707 ^c^	0.828 ^c^	0.036 ^b^	0.016 ^a^	0.451 ^c^	0.610 ^c^	0.053 ^c^	0.912 ^c^	0.432 ^c^
	*F ratio*	0.70	0.15	0.05	6.38	9.37	0.63	0.28	5.15	0.01	0.68

X_1_, X_2_, X_3_ and X_4_ represent the main or single effect of mixed soy lecithin, squalene, cholesterol and Tween80, respectively. X_1_X_2_, X_1_X_3_, X_1_X_4_, X_2_X_3_, X_2_X_4_ and X_3_X_4_ represent the interaction between mixed soy lecithin and squalene, between mixed soy lecithin and cholesterol, between mixed soy lecithin and Tween80, between squalene and cholesterol, between squalene and Tween80, and between cholesterol and Tween 80, respectively. ^a^ The most significant (*p* < 0.05). ^b^ The least significant (*p* < 0.05). ^c^ Nonsignificant (*p* > 0.05).

**Table 5:** The predicted and experimental values of the response variables of optimized liposome (5% (*w*/*w*) mixed soy lecithin (ML), 0.5% (*w*/*w*) squalene (SQ), 0.3% (*w*/*w*) cholesterol (CH) and 0.75% (*w*/*w*) Tween80 (TW80)).

Response Variables	Experimental Value	Predicted Value	Desirability
Z-average (nm) ± SD	158.31 ± 2.96	154.75	0.540
ZP (mV) ± SD	−26.84 ± 0.48	−25.88	0.943
TEAC (µM)	93.02	90.41	0.694
Particle size increase (%)	15.09	13.56	0.954
TEAC loss (%)	35.69	34.55	0.796
Composite			0.797

ZP and TEAC are zeta potential and trolox equivalent antioxidant capacity, respectively.

**Table 6:** Changes in the particle size, zeta potential (ZP) and trolox equivalent antioxidant capacity (TEAC) of the optimized squalene (SQ)-loaded and free (empty) liposomes after the storage period.

	Storage Condition	Liposome Sample	Week 0	Week 1	Week 2	Week 3	Week 4	Week 6	Week 8
Particle size (nm)	4 °C	SQ-loaded liposome	154.66 ± 4.24	–	156 ± 3.41	–	160 ± 5.73	167.66 ± 4.18	175 ± 2.24
		Empty liposome	168 ± 5.44	–	170.66 ± 3.58	–	172.66 ± 4.94	176 ± 5.13	181 ± 4.69
ZP (mV)	4 °C	SQ-loaded liposome	−24.97 ± 1.76	–	−23.98 ± 1.43	–	−23.13 ± 2.21	−21.45 ± 3.48	−18.17 ± 3.29
		Empty liposome	−21.18 ± 2.16	–	−20.17 ± 4.81	–	−19.11 ± 4.37	−18.1 ± 3.20	−17.02 ± 3.38
TEAC (µM)	45 °C	SQ-loaded liposome	93.09	90.17	79.96	65.26	49.75	–	–
		Empty liposome	46.86	39.07	31.04	24.59	19.38	–	–

Optimized squalene (SQ)-loaded liposome: (5% (*w*/*w*) mixed soy lecithin (ML), 0.5% (*w*/*w*) squalene (SQ), 0.3% (*w*/*w*) cholesterol (CH) and 0.75% (*w*/*w*) Tween80 (TW80)). Optimized squalene (SQ)-free (empty) liposomes: 5% (*w*/*w*) mixed soy lecithin, 0.3% (*w*/*w*) cholesterol and 0.75% (*w*/*w*) Tween80.

**Table 7:** Range of components of mixed soy lecithin (ML).

	Ingredients (%)
Phosphatidylcholine	19–21
Phosphatidylethanolamine	8–20
Inositol phosphatides	20–21
Other phosphatides	5–11
Soybean oil	33–35
Carbohydrates, free	2–5
Moisture	1
